# Supplementary material for: Fucoid Macroalgae Have Distinct Physiological Mechanisms to Face Emersion and Submersion Periods in Their Southern Limit of Distribution
Source: Plants (Basel). 2021 Sep 14;10(9):1892. doi: 10.3390/plants10091892 (PMC8467972; doi:10.3390/plants10091892)
Supplement: Supplementary file 1 [file plants-10-01892-s001.zip › plants-1332356-supplementary.pdf]

Supplementary Materials

Table S1. Summary of ANOVAs for chlorophyll and carotenoid contents of *P. canaliculata, A. nodosum* and *F. serratus* at low and high tide conditions in August 2017, March and August 2018.

Source	df	Chlorophylls			Carotenoids			F versus
		MS	F	*p*	MS	F	*p*	
Species (Sp)	2	0.6766	4.01	0.1107	0.1285	18.40	**0.0096**	Sp × Da
Tide (Ti)	1	1.2490	57.26	**0.0170**	0.0783	26.27	**0.0360**	Ti × Da
Date (Da)	2	0.1814	7.05	0.0026	0.0135	5.46	0.0085	Residual
Sp × Ti	2	0.1284	5.02	0.0812	0.0087	1.93	0.2590	Sp × Ti × Da
Sp × Da	4	0.1686	6.56	**0.0005**	0.0070	2.82	**0.0392**	Residual
Ti × Da	2	0.0218	0.85	0.4365	0.0030	1.20	0.3120	Residual
Sp × Ti × Da	4	0.0256	0.99	0.4229	0.0045	1.82	0.1472	Residual
Residual	36	0.0257			0.0025			
Total	36							
Cochran's test			C = 0.2264 **n.s**					C = 0.1828 **n.s.**

Sp: Species; Ti: Tide; Da: Date; n.s.: non significant.

Table S2. Summary of ANOVAs for H_2O_2 and MDA levels of *P. canaliculata, A. nodosum* and *F. serratus* at low and high tide conditions in August 2017, March and August 2018.

Source	df	H_2O_2			MDA			F Versus
		MS	F	*p*	MS	F	*p*	
Species (Sp)	2	591552.5	1.9	0.259	563.0	1.2	0.389	Sp × Da
Tide (Ti)	1	535.8	0.5	0.541	192.1	11.5	0.076	Ti × Da
Date (Da)	2	289768.4	66.7	0.000	500.9	37.8	0.000	Residual
Sp × Ti	2	2057.4	0.6	0.565	0.130	0.0	0.997	Sp × Ti × Da
Sp × Da	4	307011.9	70.7	**0.000**	467.5	35.2	**0.000**	Residual
Ti × Da	2	1003.7	0.2	0.794	16.5	1.2	0.298	Residual
Sp × Ti × Da	4	3116.6	0.7	0.585	43.9	3.3	**0.020**	Residual
Residual	36	4342.4			13.2			
Total	56							

Cochran's test	C = 0.2668 **n.s.**	C = 0.3013 **n.s.**

Sp: Species; Ti: Tide; Da: Date; n.s.: non significant.

Table S3. Summary of ANOVAs for thiols levels of *P. canaliculata, A. nodosum* and *F. serratus* at low and high tide conditions in August 2017, March and August 2018.

Source	df	Thiols			F Versus
		MS	F	*p*	
Species (Sp)	2	3.3	31.6	0.003	Sp × Da
Tide (Ti)	1	6.1	50.8	0.019	Ti × Da
Date (Da)	2	3.6	9.5	0	Residual
Sp × Ti	2	1.7	8.3	**0.037**	Sp × Ti × Da
Sp × Da	4	0.1	0.2	0.888	Residual
Ti × Da	2	0.1	0.3	0.728	Residual
Sp × Ti × Da	4	0.2	0.5	0.699	Residual
Residual	36	0.3			
Total	56				
Cochran's test			C = 0.1575 **n.s.**		

Sp: Species; Ti: Tide; Da: Date; n.s.: non significant.

Table S4. Summary of ANOVAs for proline levels of *P. canaliculata, A. nodosum, F. serratus* and at low and high tide conditions in August 2017, March and August 2018.

Source	df	Proline			F Versus
		MS	F	*p*	
Species (Sp)	2	0.017	0.7	0.529	Sp × Da
Tide (Ti)	1	0.0002	0.1	0.712	Ti × Da
Date (Da)	2	0.014	22.8	0	Residual
Sp × Ti	2	0.002	0.8	0.491	Sp × Ti × Da
Sp × Da	4	0.023	36.9	0	Residual
Ti × Da	2	0.001	2.2	0.123	Residual
Sp × Ti × Da	4	0.002	4.3	**0.006**	Residual
Residual	36	0.001			
Total	56				
Cochran's test			C = 0.2047 **n.s.**		

Sp: Species; Ti: Tide; Da: Date; n.s.: non significant.
